# Serviceability design charts for partially prestressed high-strength concrete girders

**DOI:** 10.1038/s41598-025-28287-w

**Published:** 2025-12-03

**Authors:** Shady Salem, Mohamed Abu-Krisha, Tarek Hassan, Tarek El-Hashimy

**Affiliations:** 1 Civil Engineering Department, Faculty of Engineering, The British University in Egypt, Cairo, Egypt; 2 Structural Engineering Department, Faculty of Engineering, Ain Shams University, Cairo, Egypt

**Keywords:** Serviceability design charts, Partially prestressed concrete, High-strength concrete, Cracking performance, Deflection control, Engineering, Materials science, Mathematics and computing

## Abstract

This paper presents the development of serviceability-based design charts for partially prestressed high-strength concrete (HSC) girders, with a focus on cracking and deflection performance. Partially prestressed girders, which serve as an intermediate design between fully prestressed and conventionally reinforced concrete, offer both structural efficiency and economic benefits. However, there is a notable gap in research regarding specific serviceability guidelines, particularly for controlling cracking and deflection in these girders. To address this gap, this study utilizes validated finite element models to investigate key variables, including the concrete compressive strength, prestressing forces, and non-prestressed reinforcement ratios. Parametric investigation provides insights into the impact of these factors on girder behavior under service loads, leading to the development of practical design charts. These charts allow for quick estimation of the optimal prestressing and reinforcement ratios that meet predefined serviceability limits, simplifying the design process for engineers. The proposed charts offer an accessible, efficient tool for optimizing material usage while ensuring compliance with serviceability criteria, making them highly valuable for the design of HSC girders in bridges and other infrastructures. Ultimately, this research enhances the usability of partially prestressed HSC girders, promoting more effective and economical designs in structural engineering practice.

## Introduction

Prestressed concrete bridges have gained significant attention as an attractive alternative to conventionally reinforced girders. This preference is due primarily to their superior serviceability performance, particularly in minimizing cracking and deflection. Typically, engineers utilize fully prestressed sections, which limit tensile stresses to prevent cracking and enhance durability^1^. However, research has shown that durability concerns related to cracking can also be addressed through proper reinforcement detailing and adequate concrete cover^2^.

Under service loads, partially prestressed girders typically exhibit controlled cracking with smaller crack widths, reduced crack spacing, and moderated long-term deflections compared with conventionally reinforced members, while maintaining greater ductility and energy absorption than fully prestressed girders^2–4^. Furthermore, the passive reinforcement in partially prestressed sections provides redundancy, enhancing resilience under elevated temperatures, such as during fire incidents^5,6^. Their economic viability and ability to control excessive deflection, cracking, and span limitations make them preferable to conventional reinforcement systems^2^. With these advantages, the use of partially prestressed systems becomes even more promising when combined with high-strength concrete (*HSC*), which further enhances performance.

Recent advancements in concrete technology, including the use of superplasticizers, improved mixing techniques, and advanced curing methods, have enabled the mass production of *HSC*^7^. *HSC* is characterized by higher stiffness and reduced cracking due to its linear stress-strain behavior. As a result, it has attracted significant interest for applications in beams and girders.

Many studies have demonstrated the advantages of *HSC* in structural applications. For instance, Ali and Saeed investigated the shear performance of *HSC* girders and reported that *HSC* enhances both ultimate capacity and serviceability performance^8^. Zhen-Jun et al. highlighted the stability of *HSC* capacity under high temperatures^9^, whereas El-Sayed et al. examined the flexural performance of HSC beams reinforced with hybrid-GFRP bars and demonstrated their effectiveness not only in enhancing capacity but also in integrating with fibers and limiting cracks formation^10^.

Despite extensive research on HSC, relatively few studies have focused on its application in partially prestressed girders. Roller et al. evaluated the flexural performance and ductility of bridge girders constructed with *HSC*^11,12^,while Choi et al. conducted full-scale tests on prestressed *HSC* bridge girders to investigate their flexural behavior^13^. Abdel Nour et al. proposed a dimensioning approach for partially prestressed T-section girders^14^. Nevertheless, most existing research has concentrated on fully prestressed or conventionally reinforced members, with limited investigations addressing the combined application of *HSC* and partial prestressing in girders.

Modern design standards specify serviceability limits to control deflection and cracking in reinforced concrete members^15,16^. These limits aim to protect non-structural components and ensure structural functionality^17–19^. Crack width limits are also set to address durability and reinforcement corrosion^19^. Additionally, allowable stress limits are provided to offer engineers flexibility within practical design ranges. For partially prestressed girders, these limits depend on key design parameters, such as the concrete strength, prestressing eccentricity, and reinforcement ratios, resulting in a broad spectrum of feasible design options^20^. To address this gap, the present study develops and validates numerical models for partially prestressed *HSC* girders, and proposes serviceability-oriented design charts to guide practical design applications.

## Objectives and approach

Designing partially prestressed girders often involves iterative optimization process to balance prestressing levels with serviceability requirements. This study aims to simplify this process by offering insights into the influence of key design parameters, mentioned earlier, on serviceability performance. To achieve this goal, serviceability-based design charts are developed for partially prestressed sections with various design parameter combinations. These charts are derived from a numerical database validated against experimental results from the literature^21,22^. Numerical modeling, conducted in ABAQUS using the concrete damage plasticity (CDP) model to accurately simulates concrete behavior under monotonic, cyclic loading conditions^23^.

The validation was performed on nine prestressed girder specimens with diverse concrete strengths, reinforcement ratios, and cross-sections. Subsequently, full-scale bridge girder models with three cross-sections (rectangular, I-, and T-sections) were developed to assess stress distribution and serviceability performance under realistic loading conditions. The resulting numerical database was then used to define optimal design spectra based on cracking and deflection limits, forming the basis of the proposed serviceability-oriented design charts.

## Numerical model

### Model development

**Fig. 1:**
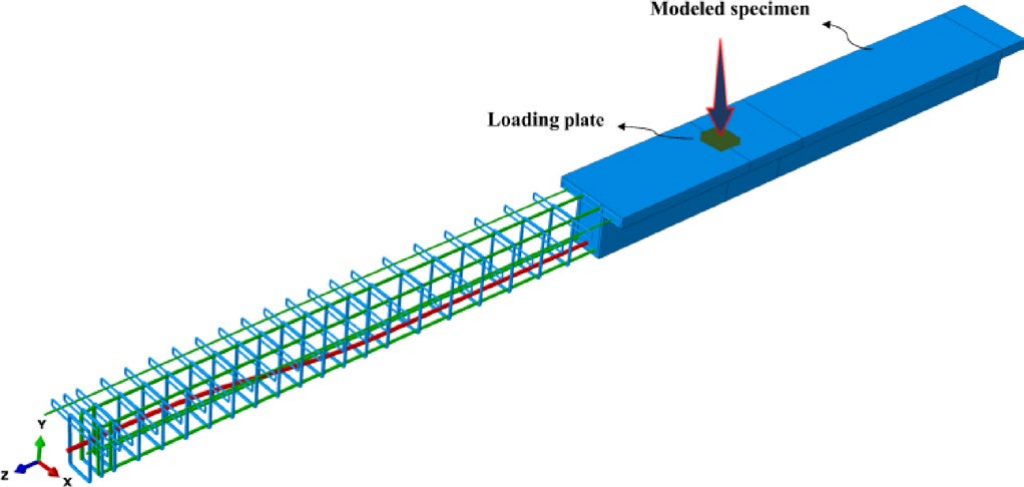
Developed three-dimensional numerical model.

The finite element software ABAQUS was used to develop three-dimensional numerical models of partially prestressed girders under quasi-static loading. As shown in Fig. 1, the concrete girders, loading plates, and supports were modelled using 3D solid deformable elements (C3D8), while discrete 3D deformable truss elements (T3D2) were used to model the longitudinal reinforcement, stirrups, and prestressing steel^14^. The interaction between reinforcements and concrete was modeled as fully bonded using embedded constraints, whereas surface-based tie constraints simulated interactions between external components such as supports and loading plates. A mesh sensitivity analysis was conducted, identifying 15 mm as the optimal mesh size for balancing accuracy and computational efficiency. Prestressing forces were applied using the temperature load method, which simulates equivalent prestressing strains. The applied temperature was calculated via Eq. (1)^24^.

1 $$^\circ C = - P/A.E.C$$

Where *C* is the coefficient of linear expansion ($\:{10}^{-5}$MPa /ºc); *P* is the effective force after losses have taken place; *E* is the modulus of elasticity in MPa, and *A* is the prestressing tendon cross-sectional area in mm^2^.

### Material modeling

Concrete behavior was modeled using the concrete damage plasticity (*CDP*) approach, which accounts for tensile cracking and compressive crushing as recommended by Drucker-Prager criteria^25^. Table 1 summarizes the key *CDP* parameters including dilation angle, stress ratio, flow potential eccentricity, and viscosity. The concrete stress-strain relationship was based on nonlinear elastic-plastic behavior, derived from the fib Model Code equations as expressed in Eq. (2) to (4). Furthermore, the nonlinear degradation of the concrete stiffness was captured through a degradation damage factor ($\:{d}_{c}$), which represents the material deterioration under loading. It was assumed that damage initiates only after the maximum stress is attained^25^. In this context, the damage factor $\:{d}_{c}$ ranges from zero (undamaged material) to one(complete loss of strength), as formulated in Eq. (5).

2 $${E}_{ci}=21.5x{10}^{3}x0.95{\left(\frac{{f}_{cm}}{10}\right)}^{1/3},{f}_{cm}={f}_{ck}+8$$

3 $$\frac{{\sigma\:}_{c}}{{f}_{cm}}=-\left(\frac{k\cdot\:\eta\:-{\eta\:}^{2}}{1+\left(k-2\right)\cdot\:\eta\:}\right)$$

4 $$\eta\:=\frac{{\epsilon\:}_{c}}{{\epsilon\:}_{cu}}\:\:\:,\:\:{\epsilon\:}_{cu}=0.0007.{{f}_{cm}}^{0.31}$$

5 $${d}_{c}=1-\raisebox{1ex}{$f$}\!\left/\:\!\raisebox{-1ex}{${f}_{cm}$}\right.$$

Where ${E}_{ci}$ is the modulus of elasticity in MPa, ${f}_{cm}$ is the mean value of compressive strength, ${f}_{ck}$ is the characteristic compressive strength in MPa, ${\epsilon\:}_{c}$ is the compressive strain and, ${\epsilon\:}_{cu}\:$is the strain at the maximum compressive stress.

**Table 1:** Flow parameters of the CDP model.

Dilation angle ($\:\phi\:$)	Eccentricity (F)	viscosity parameter ($\:\mu\:$)	$\:{K}_{c}$ (default)	$\:{f}_{bo}/{f}_{c}$ (default)
36^o^ & 41^o^	0.1	0.0001	0.667	1.16

Moreover, tension stiffening effects were incorporated to enhance the prediction of cracking behavior. The tensile behavior was considered linear elastic up to the concrete ultimate tensile strength threshold, which corresponds to the first crack (onset of cracking) of the concrete material. Later, after the formation of microcracks, the concrete tensile strength is represented by the softening branch of the curve. The ultimate tensile strength $\:\left({f}_{ctm}\right)$ was calculated using Eq. (6) as recommended by the Fib Model Code, based on the concrete compressive strength.

6 $$f\left(x\right)=\left\{\begin{array}{c}0.3\:{\left({f}_{ck}\right)}^{\raisebox{1ex}{$2$}\!\left/\:\!\raisebox{-1ex}{$3$}\right.}\:\:\:\:\:\:\:\:\:\:\:\:\:\:,\:\:Concrete\:grades\le\:50MPa\\\:2.12\text{ln}\left(1+0.1{f}_{cm}\right),\:\:Concrete\:grades\le\:50MPa\end{array}\right.$$

**Fig. 2:**
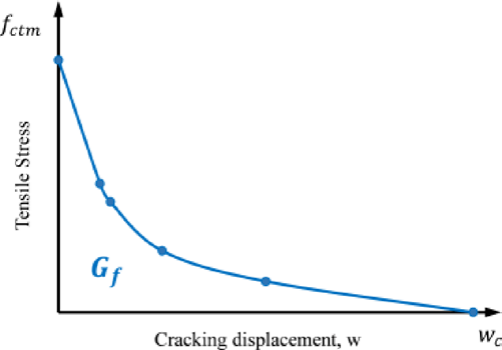
Schematic Stress-crack opening relation for uniaxial tension.

With respect to the post-peak stress-strain relation, the fracture energy $\:{(G}_{f})$, a material property defined as the amount of energy required to form a unit area of crack, was used to predict the crack development. $\:{G}_{f}$ was computed by integrating the area enclosed under the stress-cracking displacement curve^26^ as shown in Fig. 2 and defined by Eq. (7).

7 $${G}_{f}={\int\:}_{0}^{{w}_{c}}{\sigma\:}_{t}dw$$

where $\:{\sigma\:}_{t}$ is the tensile stress as a function of displacement, is the cracking displacement $\:{w}_{\:}$and $\:{w}_{c\:}$ is the maximum cracking displacement. The stress-crack displacement relationship was expressed according to Eq. 8, whereas the fracture energy and maximum cracking displacement, $\:{w}_{c\:}$, were determined using Eq. (9) and Eq. (10) respectively, as recommended by^27^.

8 $${f}_{ct}=\left(1+\left({C1\frac{w}{wc})}^{3}\right){e}^{-C2\frac{w}{wc}}-\frac{w}{wc}\left(1+{C1}^{3}\right){e}^{-C2}\right).{f}_{ctm}$$

9 $${G}_{f}=0.073\:{{f}_{cm}}^{0.18}\:\:$$

10 $${w}_{c}=5.14\frac{{G}_{f}}{{f}_{ctm}\:}$$

Where C1 = 3, C2 = 6.93, $\:w$ is the crack opening in mm, $\:{f}_{ctm}\:$is the tensile strength in MPa.

As for the reinforcement material, the conventional reinforcement was modeled using an idealized bi-linear stress-strain relationship, while the prestressing low relaxation seven-wire strands were modeled using Eq. 11.

11 $${f}_{ps}={\epsilon\:}_{ps}\left[A+\frac{B}{{\left\{1+{\left(C{\epsilon\:}_{ps}\right)}^{D}\right\}}^{\frac{1}{D}}}\right]\le\:{f}_{pu}$$

where $\:{f}_{pu}$ is the ultimate tensile strength, $\:{f}_{ps}$ is the stress corresponding to a specific strain $\:{\epsilon\:}_{ps}$ and $\:{f}_{py}$ is the yield tensile stress. The constants A, B, C, and D used in this study are summarized in Table 2. The mechanical properties of both conventional and prestressing reinforcements are presented in (Table 3)^28^.

**Table 2:** Power formula constants of 1860 MPa strands by^29^.

Strand type	$$\:\raisebox{1ex}{${f}_{py}$}\!\left/\:\!\raisebox{-1ex}{${f}_{pu}$}\right.$$	A	B	C	D
Grade 1860	0.90	887	27,613	112.4	7.360

**Table 3:** Summary of prestressing and conventional reinforcement mechanical properties^28^.

Property	Conventional reinforcement	Prestressing strands
Weight density (KN/m^3^)	7.85	7.85
Young’s modulus (MPa)	200,000	196,500
Poisson’s ratio	0.3	0.3
Yield strength (MPa)	411	1675
Yield strain	0.002	0.01
Ultimate strength (MPa)	600	1862
Ultimate strain	0.118	0.05

## Model validation

### Validation matrix

**Fig. 3:**
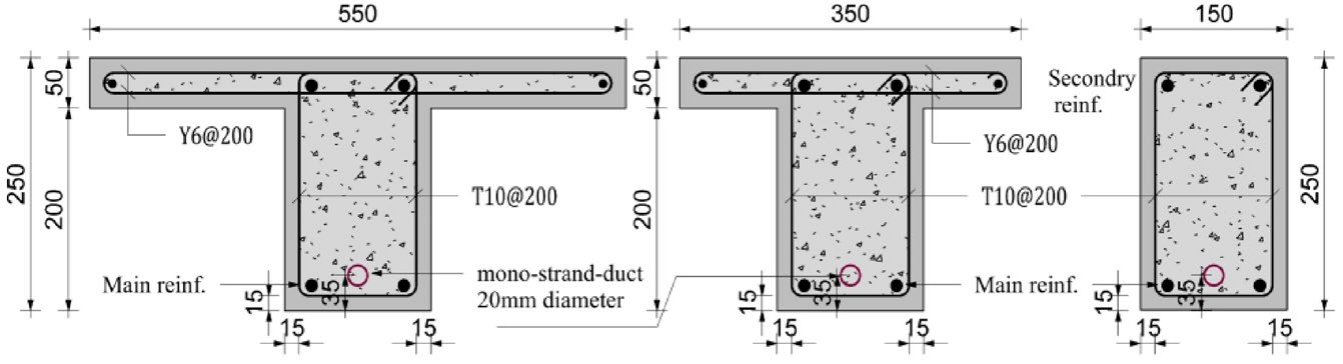
Cross section of tested girders at midspan.

The validation database was compiled from experimental studies reported in the literature for partially prestressed girders. This dataset encompasses a wide range of parameters, including concrete compressive strength, ratios of prestressing and conventional reinforcement, and variations in cross-sectional geometry, specifically rectangular and T-Sects^21,22^. The database comprises nine quarter-scale, simply supported, partially prestressed girders. Six of which are T-section girders, one with a wider flange of 550 mm width, while the remaining girders have a flange width of 350 mm, and three rectangular sections with a depth of 250 mm, as shown in Fig. 3.

Each girder has a total length of 4.8 m, with a clear span of 4.5 m, and is subjected to four-point bending with a 1.4 m spacing between the applied loads. All girders were designed with a trapezoidal cable profile to counteract the anticipated loading conditions. Table 4 summarizes the properties of the collected database, including the concrete compressive strength, prestressing and non-prestressing reinforcement ratios, and section type.

**Table 4:** Summary of the details of the girders tested by^22,30^.

Girder designation	Prestressing steel ratio (%)	Prestressing strands	Non-prestressed steel	compressive strength (MPa)	Cross section
T-85-2-2	0.43	1-0.6”	2T10	84.5	T
T-45-2-2	0.43	1-0.6”	2T10	46.5	T
T-100-2-2	0.43	1-0.6”	2T10	101.3	T
T-85-2-1	0.43	1-0.6”	2Y6	84.5	T
T-85-2-3	0.43	1-0.6”	4T10	84.5	T
R-85-1-2	0.264	1-0.5”	2T10	84.5	R
R-45-1-2	0.264	1-0.5”	2T10	46.5	R
T-85-1-2	0.264	1-0.5”	2T10	84.5	Wide-T
R-85-3-2	0.373	1-0.6”	2T10	84.5	R

### Validation parameters

For the purpose of validating the finite element (FE) models, three validation criteria were selected: failure mode, load–deflection response, and cracking response. These criteria were chosen as they provide a comprehensive representation of both global and local structural behavior and are directly comparable to typical experimental observations from girder testing. Although strain measurements are frequently reported in experimental studies, they were excluded from the present work due to the limited reliability and availability of consistent strain data across the full loading range.

#### Failure mode

**Fig. 4:**
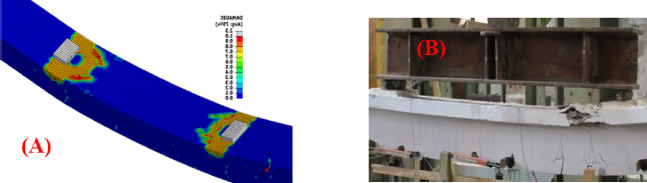
Typical concrete crushing failure of: (**A**) FE models; (**B**) Experimentally tested girders.

All the tested girders exhibited a failure mode similar to that observed in the laboratory. More specifically, seven girders failed due to crushing of the concrete in the upper compression zone within the constant bending region, as shown in Fig. 4, while two girders (T-85-2-1, T-84-1-2) failed by rupture of the prestressing strands before reaching the ultimate compressive strain. Notably, the experimental testing of these two girders was terminated at a safe load level before failure for safety precautions in the laboratory^30^.

#### Load deflection performance

**Fig. 5:**
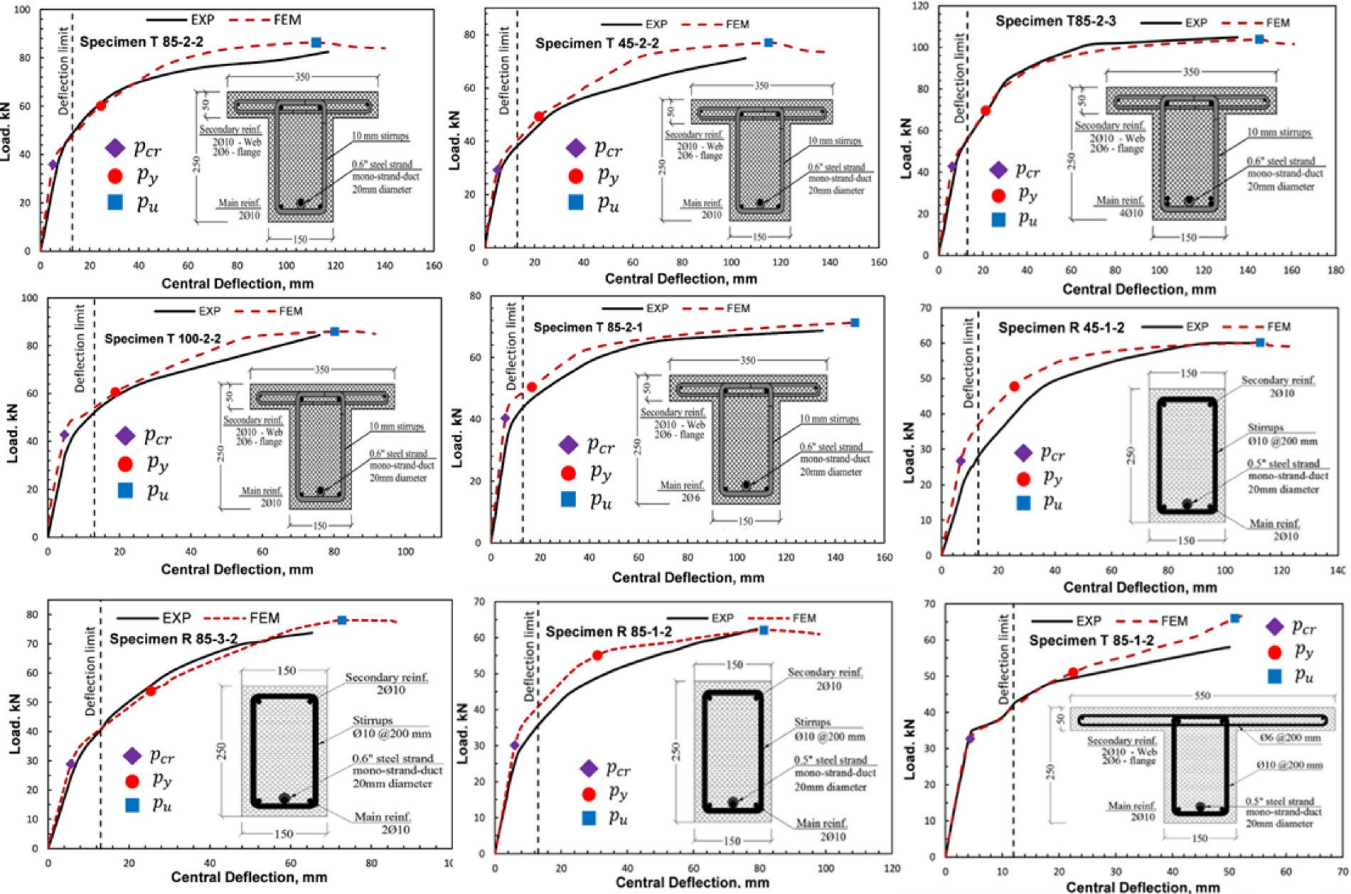
Load deflection validation.

All the developed models for the nine validation girders exhibited nonlinear load-deflection behavior that aligned well with the experimental results. The models showed good agreement with the experimental results in terms of the initial stiffness, cracking, yielding, and ultimate capacities, as depicted in Fig. 5 and summarized in Table 5. With respect to the cracking stage, the average deviation between the numerical and experimental results was 7.2% for the cracking load. The average deviations were 10.6% and 4.7% for the yielding and ultimate loads, respectively. For the ultimate deflection, the average deviation was 7.3%. Moreover, the reliability of the developed models was further assessed at the serviceability stage, defined as the point corresponding to the allowable deflection limit (span/360)^15^. The models showed an average deviation of 7.9% for the load at the allowable deflection limit.

**Table 5:** Validation of the FE against the experimentally tested results.

Girder designation	$\:{P}_{cr}\:\left(kN\right)$	$\:{\delta\:}_{allw}\:\left(kN\right)$	$\:{P}_{y}\:\left(kN\right)$	$\:{P}_{u}\:\left(kN\right)$	$\:{\delta\:}_{u}\:\left(kN\right)$	Failure mode
T85-2-2	40	47.86	67	81.52	117.05	CC
FE Model	36.86	46.56	58.25	86.33	112.3	CC
Variation (%)	-7.85	-2.71	-13.1	+ 5.90	-4.0	
T100-2-2	39.36	51.25	53	84.38	75.94	CC
FE Model	42.7	53.6	57.9	86.2	84.7	CC
Variation (%)	+ 8.50	+ 4.60	+ 9.25	+ 2.1	+ 11.5	
T45-2-2	29.87	36.2	44	70.08	105.8	CC
FE Model	28.86	40.56	49.7	77.22	115.4	CC
Variation (%)	-3.38	-12.0	-13.0	+ 10.3	+ 9.0	
T85-2-1	37.89	42.78	46.31	68.68	134.94	PR
FE Model	41.1	47.6	48.8	71.3	148.12	PR
Variation (%)	+ 8.47	+ 11.3	+ 5.3	+ 3.81	+ 9.80	
T85-2-3	44.64	55.06	88.34	104.86	135.52	CC
FE Model	41.7	55.65	74.9	103.8	145.6	CC
Variation (%)	-6.50	+ 1.0	-15.2	-1.10	+ 7.45	
R-85-1-2	27	35.27	–	61.3	79.2	CC
FE Model	30.85	39.7	54.4	62.72	82.25	CC
Variation (%)	+ 10.55	+ 12.60	–	+ 2.3	+ 3.85	
R-45-1-2	25	27.33	–	60	109.4	CC
FE Model	27.85	32.58	48.98	60.76	113.4	CC
Variation (%)	+ 11.40	+ 19.2	–	+ 1.25	+ 3.65	
T-85-1-2	35	43	–	58.02	50	PR
FE Model	32.64	41.6	48.96	66.24	52.4	PR
Variation (%)	-6.75	-3.25	–	+ 13.8	+ 4.80	
R-85-3-2	30	39.64	–	73.7	65.3	CC
FE Model	30.42	40.8	52.44	78	72.7	CC
Variation (%)	+ 1.4	+ 2.9	–	+ 5.83	+ 11.33	

$\:{P}_{cr}$ represent the load at cracking initiation, $\:{P}_{u}\:$ is the ultimate load and $\:{\delta\:}_{u}$ is the corresponding deflection, $\:{\delta\:}_{allw}$ is the permissible deflection load at 12.5 mm, $\:{P}_{y}$ is load of tension reinforcement yielding and CC = concrete crushing while PR = prestressing strands rupture.

#### Cracking performance

**Fig. 6:**
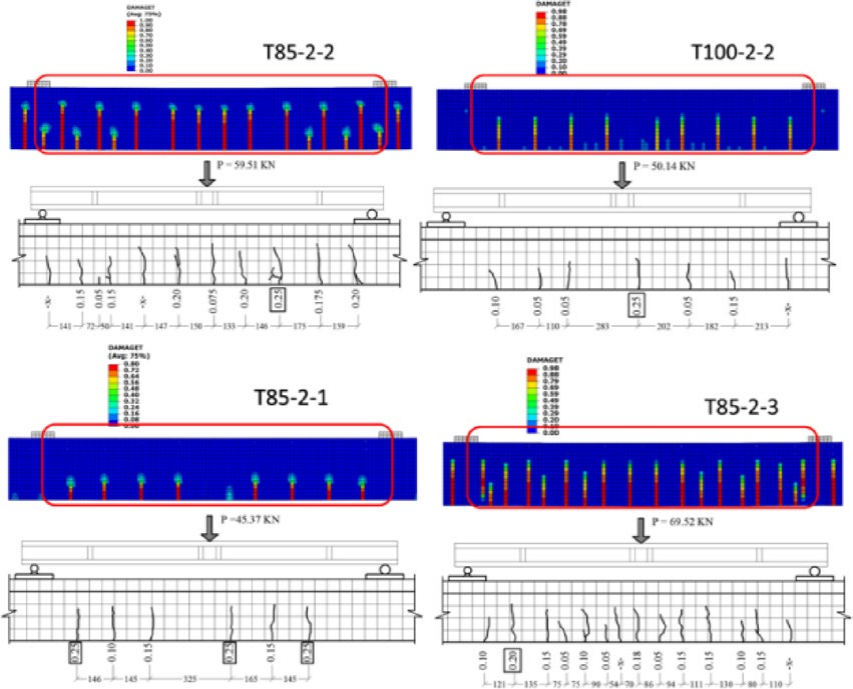
Crack pattern validation.

The cracking assessment included the crack propagation pattern and the average crack width. The numerical model showed a similar trend as that observed within the experimental testing, where the cracks propagated in a vertical pattern initiated near the stirrups location, as reported within the experimental database and shown for the sample of the validation girders. Figure 6 shows a comparison between the experimentally recorded crack patterns with the FE model predictions for the validation girders at the stabilized cracking stage, where primary distinguished cracks are visible on the concrete surface within the constant moment zone, as reported by the experimental database^30^. Each red line represents a primary surface crack, while small secondary cracks, located between them are associated with the bond interaction between concrete and reinforcement.

For the crack width, a simplified approach was proposed and validated to infer the crack width from the numerical model. The proposed method provides an acceptable prediction of the mean crack width and uses the fracture energy to estimate the crack width as proposed^31^. The method relates the equivalent crack width with the observed tensile damage variable for each element via the adopted crack propagation model as depicted in Fig. 2. It is worth mentioning that the proposed approach has several limitations based on the assumptions used. These assumptions can be summarized as follows:

- Modeling the concrete as a homogeneous material results in symmetric crack patterns.
- Each finite element meshes to have a single dominant crack.
- Cracks initiate in each element once the stress reaches the concrete tensile strength, and tensile damage develops thereafter.
- The average crack width prediction has high accuracy before reaching two-thirds of the reinforcement yielding load^31^.
- The proposed method is sensitive to the accuracy of the fracture energy calculation.

**Fig. 7:**
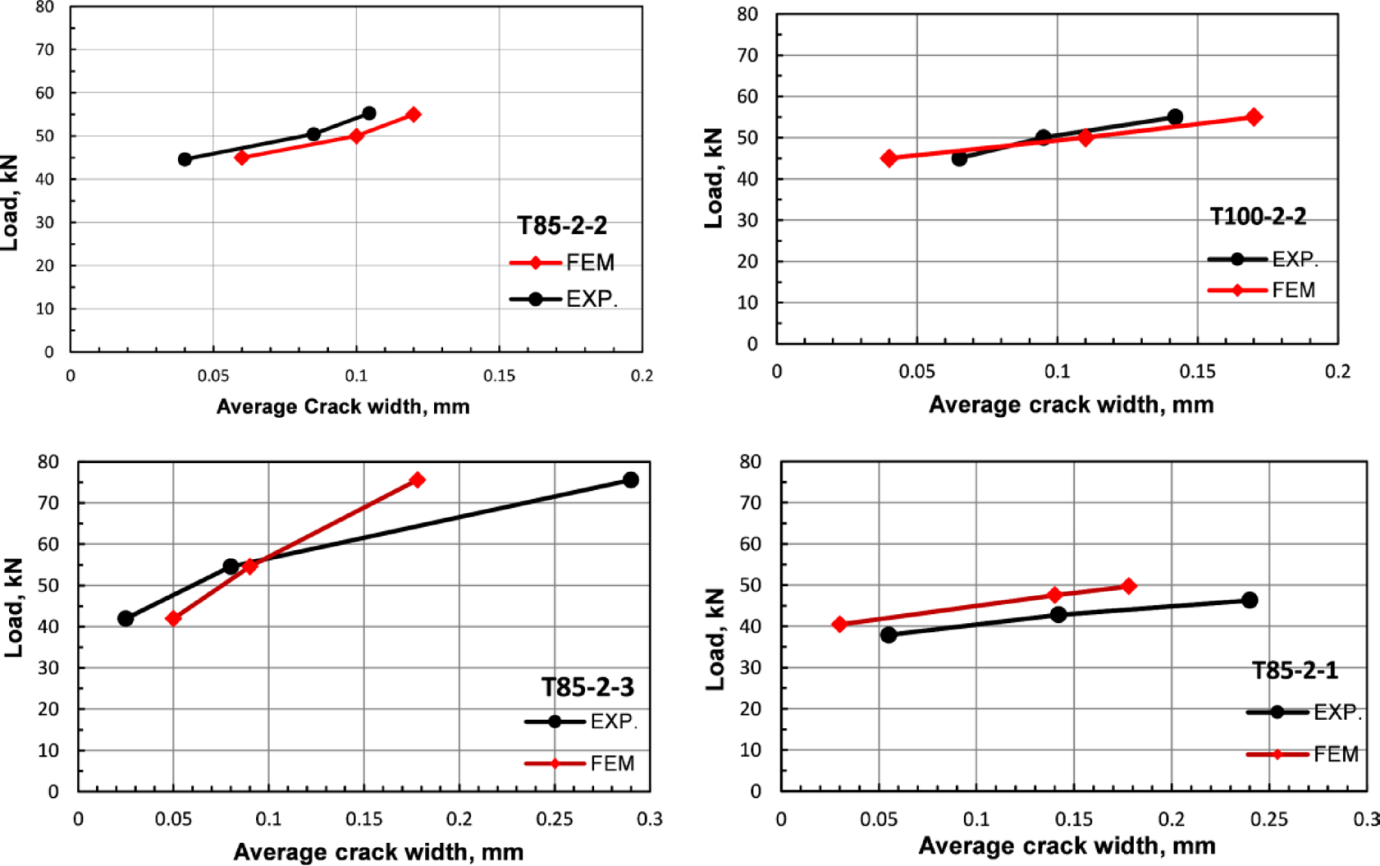
Average crack width validation.

Figure 7 shows a comparison between the experimentally measured average crack width and that obtained numerically for a sample of the validation girders. The validation shown in Fig. 7 reveals the good agreement (within 10% deviation) between the model and experimental results, especially within the allowable code limits of 0.15 mm (i.e., intermediate crack limit between the indoor and outdoor structures)^15^.

## Parametric study

### Parametric database

**Fig. 8:**
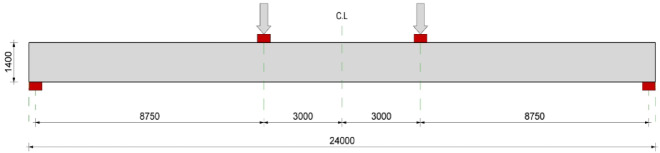
Elevation view for the modeled girders.

**Fig. 9:**
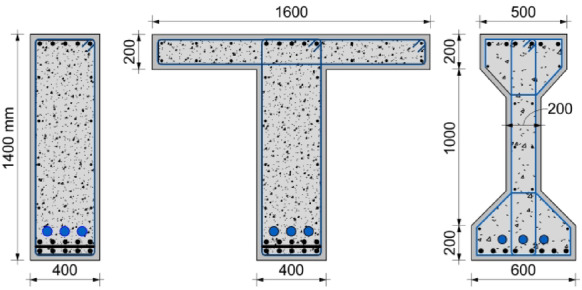
Geometric shapes of modelled girders.

This section presents a numerical parametric study conducted on full-scale partially prestressed girders to examine the influence of concrete compressive strength, as well as prestressed and non-prestressed reinforcement ratios across various cross-sectional configurations. The full-scale model was developed by scaling (5:1) the validated model into realistic bridge girder dimensions. All modeled girders had a length of 24.0 m and a clear span of 23.5 m under simple support conditions. The girders were subjected to a four-point bending scheme with a constant shear span of 8.75 m as depicted in Fig. 8. In this context, all girders in parametric study were modeled using their full-length geometry, without employing symmetry assumptions, to capture the complete structural response. The parametric database comprises three series of cross-sections: rectangular, T, and I sections as shown in Fig. 9. The properties of prestressed and non-prestressed reinforcement were assumed to be consistent with those used in the validation process. Prestressing losses were manually calculated using the ACI 423.10 standards^32^. The prestressed strands were positioned along the beam span with a polygon profile of curved edges, similar to the validation models, with an eccentricity of 580 mm for rectangular and I-section girders and an eccentricity of 760 mm for T-section girders, ensuring minimum cover for the prestressing ducts as shown in Fig. 9. It worth mentioning that the prestressing wires (up to 22 strands) were modeled individually according to a practical strand pattern, ensuring that the influence of distribution was fully captured.

Within the developed parametric database, the concrete compressive strength varied between 40 MPa and 100 MPa (specifically, 40, 80, 90, and 100 MPa). The prestressing reinforcement ratios ranged from 0.35% to 0.60%, whereas the non-prestressing steel ratios varied between 0.22% and 1.25%. To ensure a meaningful comparison, all models were designed to encompass different combinations of these parameters while maintaining similar ultimate capacities. In other words, the design parameters were adjusted to achieve comparable ultimate capacity across the models. As a result of this methodological approach, twenty-four finite element (FE) models were developed. The influence of concrete compressive strength and variations in prestressing and non-prestressing steel were specifically analyzed through the behavior of rectangular girders, whereas the effects of different cross-sectional configurations (T and I sections) were evaluated under a constant compressive strength of 80 MPa.

To facilitate the interpretation of the findings, the developed models were systematically named according to their design parameters. The naming convention consisted of four components: the first letter denoting the girder cross-section (R, T, or I), a numerical value indicating the concrete compressive strength (40, 80, 90, or 100 MPa), followed by two numerical values representing the prestressed and non-prestressed reinforcement ratios. For example, the designation “T-80-0.6-0.22” corresponds to a T-section girder with a concrete compressive strength of 80 MPa, a prestressing ratio of 0.6%, and a conventional reinforcement ratio of 0.22%. Table 6 summarizes all design parameters within the parametric database. The selected reinforcement ratios were chosen to reflect realistic values commonly employed in design practice, ensuring that the conclusions drawn from this study remain relevant and applicable to engineering practice. It is also noteworthy that the compression reinforcement was kept constant at 0.15% of the cross-sectional area for all models, given its minimal expected influence on the behavior of partially prestressed girders^33^.

**Table 6:** Specimens of parametric database.

Specimen	Group	Compressive strength (MPa)	Tension reinforcement	Prestressed strands	Compression reinforcement
R-80-0.6-0.22	R1	80	6T16	22 − 0.60”	4T16
R-80-0.5-0.6	R1	80	7T25	18 − 0.60”	4T16
R-80-0.4-1.0	R1	80	7T32	15 − 0.60”	4T16
R-80-0.35-1.24	R1	80	14T25	13 − 0.60”	4T16
R-40-0.6-0.22	R2	40	6T16	22 − 0.60”	4T16
R-40-0.5-0.6	R2	40	7T25	18 − 0.60”	4T16
R-40-0.4-1.0	R2	40	7T32	15 − 0.60”	4T16
R-40-0.35-1.24	R2	40	14T25	13 − 0.60”	4T16
R-90-0.6-0.22	R3	90	6T16	22 − 0.60”	4T16
R-90-0.5-0.6	R3	90	7T25	18 − 0.60”	4T16
R-90-0.4-1.0	R3	90	7T32	15 − 0.60”	4T16
R-90-0.35-1.24	R3	90	14T25	13 − 0.60”	4T16
R-100-0.6-0.22	R4	100	6T16	22 − 0.60”	4T16
R-100-0.5-0.6	R4	100	7T25	18 − 0.60”	4T16
R-100-0.4-1.0	R4	100	7T32	15 − 0.60”	4T16
R-100-0.35-1.24	R4	100	14T25	13 − 0.60”	4T16
T-80-0.6-0.22	T	80	7T18	32 − 0.60”	6T16
T-80-0.5-0.6	T	80	6T32	28 − 0.60”	6T16
T-80-0.4-1.0	T	80	10T32	22 − 0.60”	6T16
T-80-0.35-1.24	T	80	12T32	19 − 0.60”	6T16
I-80-0.6-0.22	I	80	5T16	18 − 0.60”	6T12
I-80-0.5-0.6	I	80	7T22	15 − 0.60”	6T12
I-80-0.4-1.0	I	80	9T25	12 − 0.60”	6T12
I-80-0.35-1.24	I	80	7T32	10 − 0.60”	6T12

### General observations

**Fig. 10:**
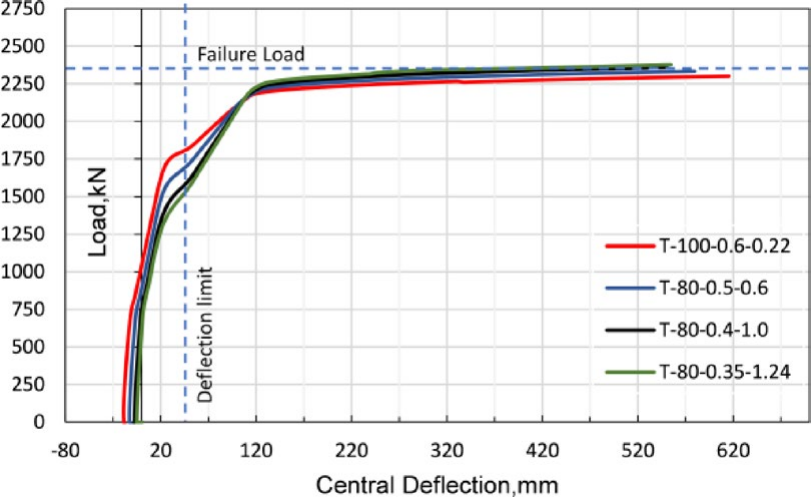
Load deflection curves for the T-section girders series.

All developed models exhibited the typical load-deflection response of partially prestressed girders. Prior to the application of external loading, the girders demonstrated an initial upward camber, followed by a linear elastic response typical of concrete behavior up to the cracking threshold. Beyond this limit, a progressive reduction in stiffness was observed until reaching the ultimate load. This behavior is illustrated in Fig. 10, which presents the load-deflection curves for a sample of the modeled T-Section girders. From Fig. 10, it is evident that the initial design of the developed models was sufficiently robust, achieving an ultimate load capacity that was consistent across different cross-sections, with a variation of approximately ± 5.0%.

### Effect of concrete compressive strength

**Fig. 11:**
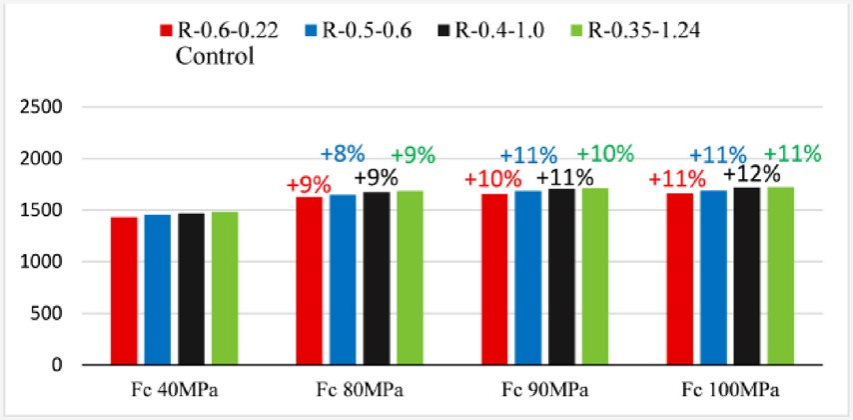
R-section series specimens failure loads.

The influence of concrete compressive strength on the structural performance of partially prestressed girders was examined using rectangular cross-section datasets. The compiled dataset encompasses a wide spectrum of concrete compressive strengths, from normal to high-strength concrete (40–100 MPa) (i.e., within the validated range presented). In terms of ultimate capacity, utilizing high-strength concrete as opposed to normal-strength concrete can result in an average increase of approximately 9% across the tested rectangular cross-section configurations (when increasing the concrete compressive strength from 40 MPa to 80 MPa), as illustrated in Fig. 11. However, within the high-strength concrete range, the observed improvement becomes marginal, with an increase of only 1–2% when transitioning from 80 MPa to 100 MPa. This limited enhancement is primarily attributed to a shift in the dominant failure mode from concrete crushing to prestressed steel rupture. It is important to note that while these enhancement ratios are specific to the investigated sections, variations in design parameters are not expected to significantly alter the overall observed trend.

**Fig. 12:**
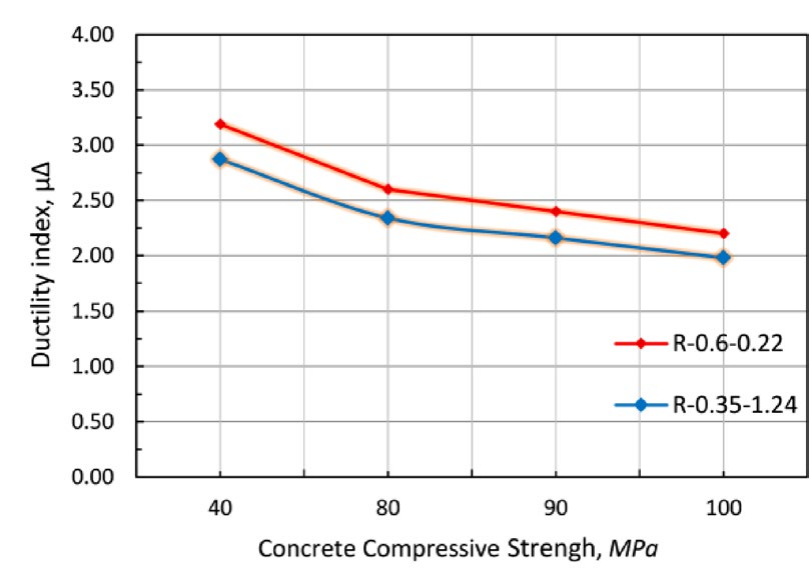
Variation of deflection ductility with compressive strength.

In terms of ductility (i.e., the ratio between the ultimate deformation and the deformation at reinforcement yielding), yielding is defined as the point at which the axial stresses in the reinforcement truss elements approach the yielding stress. The effect of concrete compressive strength on ductility was examined for two different combinations of prestressing and non-prestressing steel ratios (0.6–0.22 and 0.35–1.24). The analysis revealed a consistent inverse relationship between concrete compressive strength and ductility for both steel ratio combinations, as illustrated in Fig. 12. Specifically, increasing the concrete compressive strength from 40 MPa to 80 MPa and 100 MPa resulted in an average reduction in ductility of approximately 23% and 45%, respectively, for both investigated cases.

### Effect of prestressed and non-prestressed reinforcement ratios

**Fig. 13:**
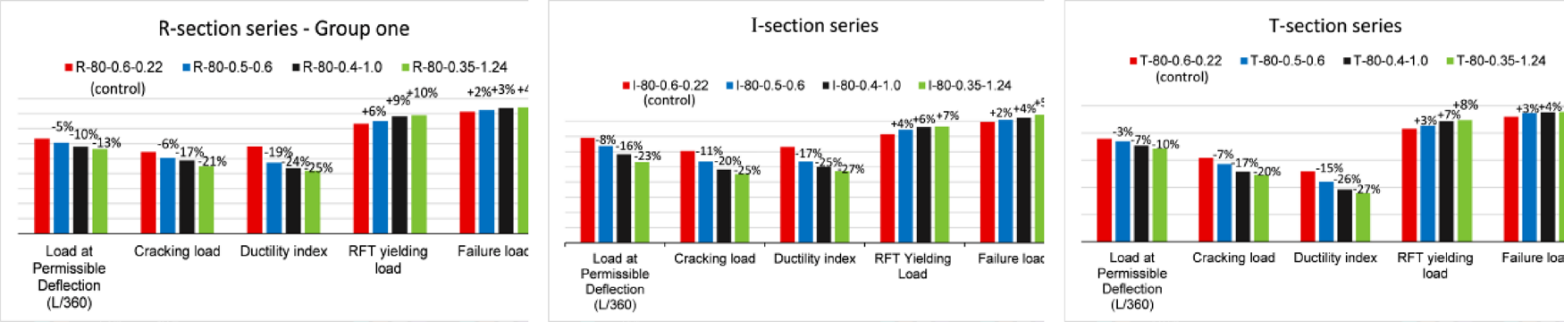
Effect of prestressed and non-prestressed reinforcement ratios on tested specimens.

Based on the developed parametric investigation, it was noticeable that the behavior of all the considered sections followed a similar trend regarding the variation of prestressed and non-prestressed ratios. The structural response was evaluated across multiple parameters, including cracking load, yielding load of non-prestressed reinforcement, and ductility. Additionally, to assess performance at the serviceability stage, comparisons were made based on the allowable deflection load limit specified in ACI 318^28^. As a general trend, reducing the prestressed reinforcement ratio while increasing the non-prestressed reinforcement ratio led to a considerable reduction in the cracking load and service load (i.e., at the allowable deflection limit) and ductility, as illustrated in Fig. 13. Where Fig. 13 demonstrates the influence of variation in prestressed and non-prestressed reinforcement ratios while maintaining the same ultimate capacity and concrete compressive strength (80 MPa) for the three investigated cross sections (i.e., rectangular, T, and I sections).

As a general trend, decreasing the prestressing steel ratio while increasing the non-prestressed steel ratio led to a significant reduction in cracking load, working load (at the allowable deflection limit), and ductility, as shown in Fig. 13. Specifically, reducing the prestressing reinforcement ratio from 0.6 to 0.35 resulted in a 20–25% decrease in cracking load and a 10–23% reduction in working load, depending on the cross-section considered. Conversely, variations in the prestressed reinforcement ratio led to a 7–10% increase in the load required for the yielding of non-prestressed steel. Consequently, ductility decreased by approximately 25–27%, depending on the cross-section under investigation.

This behavior can be attributed to the increased contribution of non-prestressed steel in resisting the prestressing force. Although this observation contrasts with the widely held assumption that ductility has a direct relationship with the non-prestressed reinforcement ratio, this discrepancy arises from the interdependent relationship between prestressed and non-prestressed steel, which is necessary to maintain the ultimate capacity of the section.

### Effect of the cross-section shape

The influence of cross-section shape on the structural behavior of partially prestressed HSC girders was examined by analyzing three different section types: rectangular, T, and I sections. All models were developed using the same concrete compressive strength of 80 MPa. Each section category consisted of four models, each with varying combinations of prestressed and non-prestressed reinforcement steel ratios. While the models within each category were designed to achieve comparable ultimate capacities, the ultimate capacities varied across the different section types due to differences in compressive flange width and the resulting section prestressing eccentricity. Therefore, this analysis highlights both the absolute effect of varying steel ratios within each section type and the relative influence of cross-section shape using normalized parameters (i.e., values normalized against ultimate capacity).

**Fig. 14:**
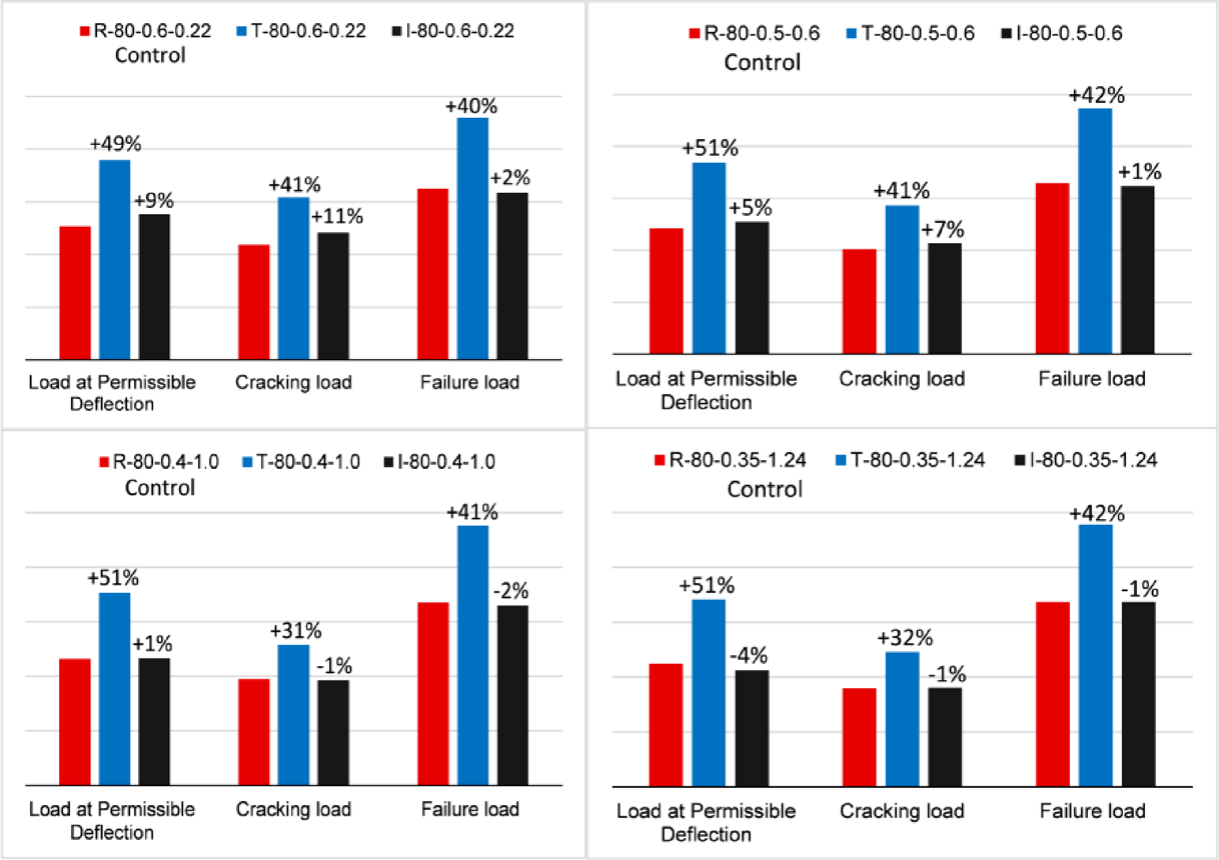
Comparison between cross section types.

The results of the T-section models indicate a substantial improvement in serviceability performance compared to the rectangular section. Specifically, for a prestressing ratio of 0.6%, the cracking load and load at permissible deflection increased by 41% and 49%, respectively. At a prestressing ratio of 0.5%, serviceability performance remained significantly enhanced, with increases of 41% and 51% in cracking load and load at permissible deflection, respectively. However, for specimens with lower prestressing ratios (0.40% and 0.35%), the improvement in cracking load was reduced to 31%, although the enhancement in load at permissible deflection remained notable, reaching 51%. Additionally, the failure load increased by an average of 41% compared to the rectangular section, as illustrated in Fig. 14. It is also important to highlight that the presence of a wider HSC flange in the T-section series altered the failure mechanism. Specifically, the failure mode shifted, leading to the rupture of prestressing strands before the concrete attained its maximum compressive strain.

Similarly, the comparative analysis of I-section girders and rectangular (R-section) girders showed an overall enhancement in terms of cracking and load at permissible deflection for all the considered reinforcement combinations. For example, the investigation at a prestressing ratio of 0.6% showed an increase in the cracking load by 11% and by 9% for the load at permissible deflection. Whereas at a prestressing ratio of 0.5%, the I-section demonstrates a marginal improvement in serviceability performance compared to the R-section, with cracking load increasing by 7% and permissible deflection load improving by 5%. However, at lower prestressing ratios (0.4–0.35%), the serviceability performance of the I-section becomes comparable to or slightly lower (by approximately 4%) than that of the R-section. In terms of ultimate capacity, both sections exhibit nearly identical values. From a general design perspective, for I-sections, a decrease in the effective prestressing force significantly impacts serviceability performance. Even when conventional (non-prestressed) reinforcement is increased to maintain the same ultimate capacity, the reduction in effective prestressing force results in diminished serviceability characteristics.

## Proposed design charts

**Fig. 15:**
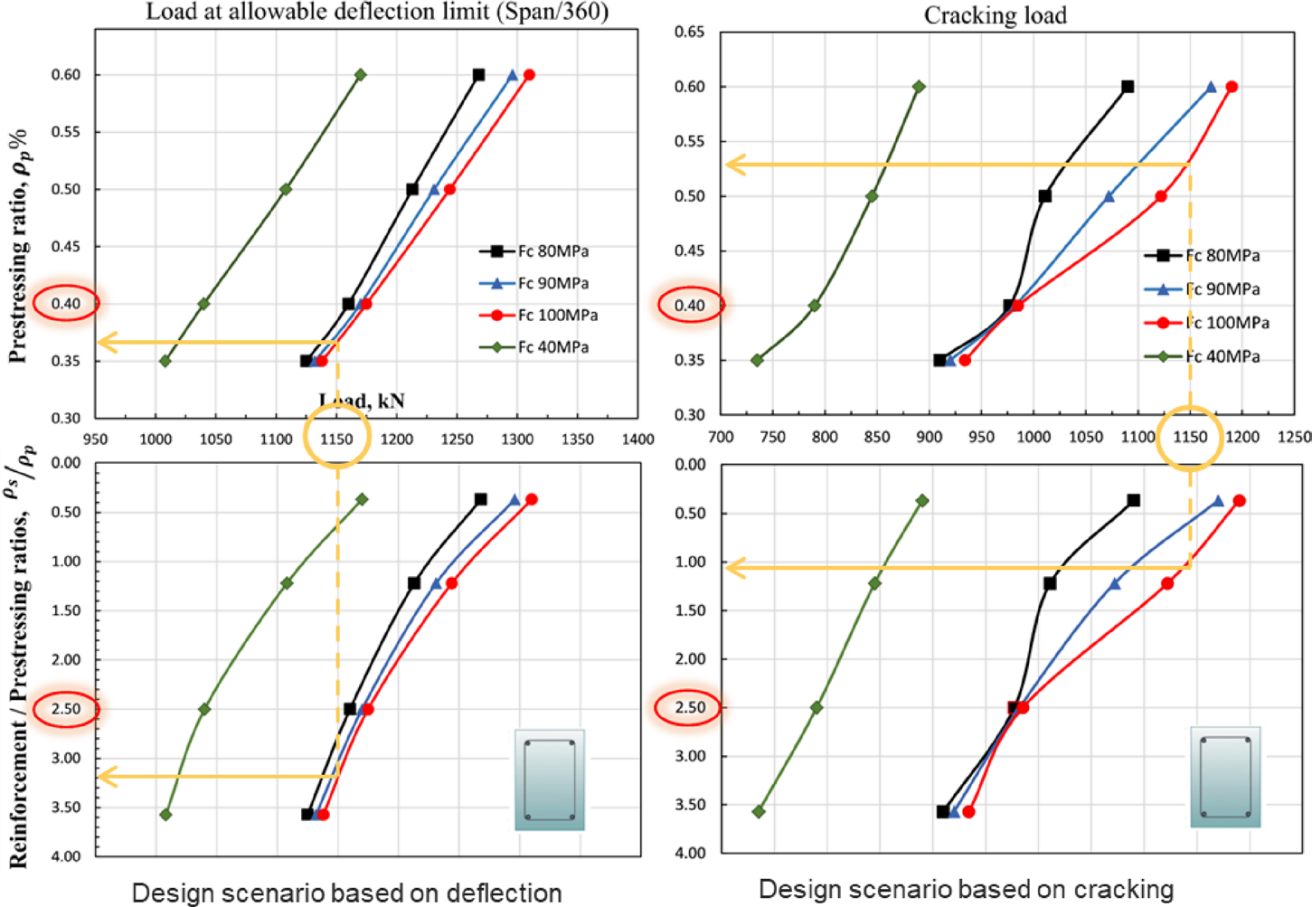
Proposed design charts for the modeled systemic girders.

This section presents the development of serviceability-based design charts for partially prestressed high-strength concrete (HSC) girders. The developed database was further used to derive schematic design charts that aimed at assisting practitioners in optimizing the combination of prestressed and non-prestressed steel while adhering to allowable deflection and cracking limits. The schematic design charts targets to provide practical framework for designers to determine the optimal reinforcement ratios while considering the most critical serviceability condition, whether deflection or cracking. The design process involves aligning the targeted working design load, selecting the appropriate concrete compressive strength, and subsequently determining the optimal ratios of prestressing and conventional reinforcement. Figure 15 demonstrates compiled charts for the design of rectangular partially prestressed girders considering the range investigated concrete compressive strength (from 40 to 100 MPa). Figure 15 is composed of four sub-figures where the upper two sub-figures demonstrate the minimum prestressing ratio to reach the considered allowable deflection and cracking, whereas the lower two sub-figures show their corresponding conventional reinforcement ratio against the prestressing reinforcement. For instance, if the design load is set at 1150 kN, the prestressing reinforcement ratio should be 0.37 and the conventional reinforcement-to-prestressing reinforcement ratio should be 3.8 to satisfy the deflection limit (span/360). However, if cracking control (0.15 mm crack width) is prioritized, the corresponding values should be 0.53 and 1.48, respectively. Additionally, the developed design charts identify a critical threshold beyond which increasing concrete compressive strength no longer significantly enhances serviceability performance. This insight aids engineers in value engineering by facilitating the selection of cost-effective and structurally efficient design solutions. Although the charts are presented here for rectangular girders, the underlying model has already been validated against T-section girders; extension to other cross-sections will need further parametric investigations tailored to the desired geometry.

**Fig. 16:**
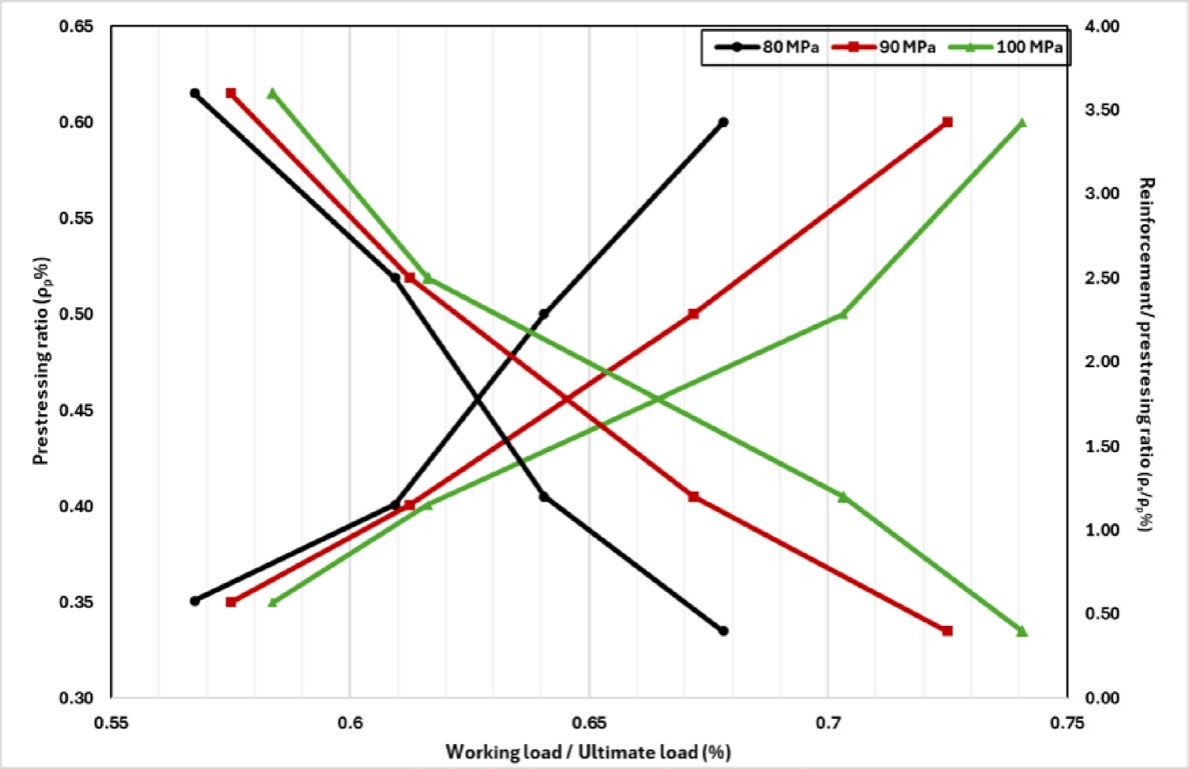
Normalized severability-based design charts for rectangular partially prestressed HSC girders.

The design charts shown in Fig. 15 represent the minimum requirements to achieve serviceability within the investigated ranges and girder dimensions. Beyond these bounds, the curves should not be directly applied, as the proposed geometric dimensions may no longer be valid. To address this limitation, generalized normalized curves are introduced in Fig. 16 to extend the applicability of the recommendations. Figure 16 depicts the influence of prestressing variation and concrete compressive strength on the minimum prestressing ratio to fulfill both the considered cracking limit (0.15 mm) and the deflection limit (span/360). Figure 16 shows that as long as the ratio of working to the ultimate load increases, the prestressing force should increase. This observation is backed to the role of prestressing force to arrest and resist cracking. In other words, the prestressing force is a dominating design factor as the ratio of the working load to the ultimate load increases. Moreover, Fig. 16 also demonstrates that the variation of concrete compressive strength is an influencing parameter as long as the working against the ultimate load increases above 61%. For example, increasing the concrete compressive strength from 80 to 100 MPa while targeting working load of 60% of the ultimate capacity can reduce the minimum prestressing ratio by 4%, whereas the same increase while targeting working load of 65% reduces the minimum prestressing ratio by 16%. To illustrate the application of these curves, consider a girder with concrete compressive strength of 90 MPa and a working-to-ultimate load ratio of 65%. From Fig. 16, the minimum prestressing ratio should be greater than or equal 0.46% of the concrete cross-section. Moreover, the non-prestressed reinforcement should be double the prestressed steel to simultaneously satisfy both the deflection and cracking thresholds. This example highlights how the normalized charts can guide designers in determining reinforcement ratios under practical design conditions.

## Conclusions

This study developed practical, serviceability-based design charts for partially prestressed high-strength concrete (HSC) girders. These charts address a critical gap in structural engineering, providing engineers with accessible tools to optimize girders performance under service loads while maintaining compliance with deflection and cracking limits. This paper presents a detailed description of the numerical model development and extensive validation. The developed models were validated against nine experimentally tested partially prestressed concrete girders. The numerically predicted stress-crack analysis results showed good agreement with the experimentally measured crack widths with deviations within 10%.

The results of the parametric study demonstrated that the serviceability performance of partially prestressed high-strength concrete girders is highly sensitive to the balance between prestressing and non-prestressed reinforcement. Reducing the prestressing ratio while increasing conventional reinforcement leads to a measurable decline in allowable deflection capacity and ductility, though the effect varies with the cross-sectional configuration. These relationships highlight the importance of maintaining an optimal reinforcement balance to achieve adequate serviceability performance without compromising ultimate strength. The observed interaction between prestressing level and conventional reinforcement provides valuable guidance for practical design, supporting the selection of efficient reinforcement combinations that satisfy both deflection and cracking limits.

The presented validation established the foundation for developing design charts, which guide the selection of optimal prestressing and reinforcement ratios for varying material strengths and serviceability criteria. Furthermore, the study identified a critical threshold beyond which increasing the concrete compressive strength provides diminishing improvements in serviceability performance. This finding offers valuable guidance for value engineering, enabling designers to achieve an effective balance between structural performance and cost efficiency.

Although long-term losses were considered in developing the proposed design charts, long-term deflection effects and different serviceability limit states, such as vibration, can be considered in future studies to reach comprehensive design charts.

In conclusion, this study makes a significant contribution to the field of structural engineering by providing practical tools for optimizing the design of partially prestressed HSC girders. While limitations remain, the insights and methodologies present form a strong foundation for further advancements. By addressing long-term performance and real-world variability, future research can ensure the continued relevance and efficacy of these tools, promoting efficient and sustainable structural designs in the construction industry.

## Data Availability

The datasets used and/or analyzed during the current study available from the corresponding author on reasonable request.
